# Exploitation of Insect Vibrational Signals Reveals a New Method of Pest Management

**DOI:** 10.1371/journal.pone.0032954

**Published:** 2012-03-21

**Authors:** Anna Eriksson, Gianfranco Anfora, Andrea Lucchi, Francesco Lanzo, Meta Virant-Doberlet, Valerio Mazzoni

**Affiliations:** 1 The Istituto Agrario di San Michele all’Adige Research and Innovation Centre, Fondazione Edmund Mach, San Michele all'Adige, Italy; 2 Department C.D.S.L., Section of Agricultural Entomology, University of Pisa, Pisa, Italy; 3 Department of Entomology, National Institute of Biology, Ljubljana, Slovenia; Ghent University, Belgium

## Abstract

Food production is considered to be the main source of human impact on the environment and the concerns about detrimental effects of pesticides on biodiversity and human health are likely to lead to an increasingly restricted use of chemicals in agriculture. Since the first successful field trial, pheromone based mating disruption enabled sustainable insect control, which resulted in reduced levels of pesticide use. Organic farming is one of the fastest growing segments of agriculture and with the continuously growing public concern about use of pesticides, the main remaining challenge in increasing the safety of the global food production is to identify appropriate alternative mating disruption approaches for the numerous insect pests that do not rely on chemical communication. In the present study, we show for the first time that effective mating disruption based on substrate-borne vibrational signals can be achieved in the field. When disruptive vibrational signals were applied to grapevine plants through a supporting wire, mating frequency of the leafhopper pest *Scaphoideus titanus* dropped to 9 % in semi-field conditions and to 4 % in a mature vineyard. The underlying mechanism of this environmentally friendly pest-control tactic is a masking of the vibrational signals used in mate recognition and location. Because vibrational communication is widespread in insects, mating disruption using substrate vibrations can transform many open field and greenhouse based farming systems.

## Introduction

For many insects, species-specific sex pheromones are essential in bringing together potential partners [1] and an early realization of the potential for exploiting chemical signals for pest control has led to a plethora of research and application through the last 50 years [2]-[4]. Today disruption of chemical communication is an integral part of pest management in several important crops worldwide [3]-[5]. However, numerous major insect pests do not rely on long-range chemical communication, most notably leafhoppers and planthoppers [6]-[8] that comprise more than 30,000 species [9]-[10]. In these insects mate recognition and localization of the partner are mediated exclusively via substrate-borne vibrational signals [11] and their populations are currently managed primarily by insecticide treatments. Surprisingly, although males use special species-specific disruptive vibrational signals to interfere with the courtship of rivals [12]-[13], mating interruption by induced vibrations has been rarely considered even from a theoretical viewpoint and there has been virtually no research on how to exploit this common insect communication channel [14] as a tool for pest control [4], [15].

Here we present the first implementation of mating disruption based on substrate-borne vibrations. The leafhopper *Scaphoideus titanus* Ball (Hemiptera: Cicadellidae), a vector of a lethal grapevine disease Flavescence dorée, was chosen as a model pest species. In Europe Flavescence dorée is a quarantine disease and compulsory measures to manage vector populations and prevent the spread of the disease include large-scale insecticide treatments [16]. In sexual communication of *S. titanus* a stable male-female vibrational duet is essential for successful localization of the female and, consequently, for copulation [13], [14], [17]. Because the initial step in pair formation of *S. titanus* is an emission of male calling signals [13], [17], we first analyzed the velocity characteristics of these vibrational signals in semi-field conditions, by applying pre-recorded calls to one leaf of the grapevine plants that were later used for mating disruption tests. Next, we established whether disruptive vibrational signals can be applied to several grapevine plants simultaneously and whether under such circumstances these signals would mask male calls. Finally, we assessed copulation success of *S. titanus* in the presence of disruptive signals under simulated semi-field conditions (potted plants) and in a vineyard with mature, field growing grapevine plants. By testing transmission of male calling signals on different plant parts, we aimed to establish the sensitivity of mating signals in order to adjust the power of the mating disruption signals into effective species-specific masking signals (disturbance noise) [13]. An electromagnetic shaker was used to vibrate the wire with disruptive signal that was transmitted as substrate vibrations to the plants in both potted and fully mature field grapevine plants.

## Results

### Semi-field

**Figure 1:**
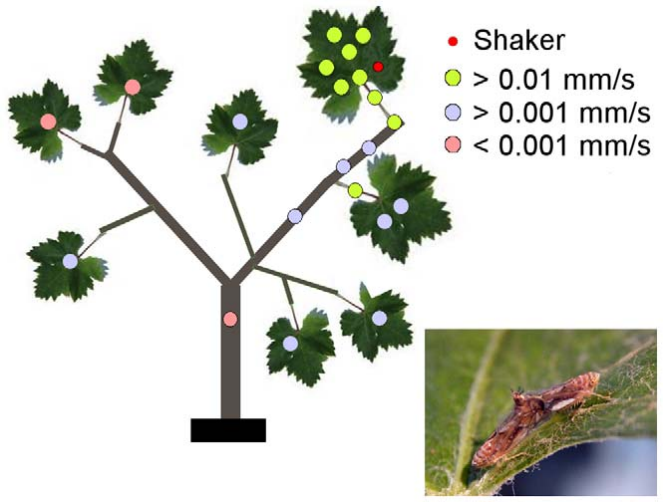
Transmission of MCS through a grapevine plant. The uppermost leaf of potted grapevine plants was vibrated with male calling signal (red dot, Shaker). The intensity of vibrational signals was measured at several points along the grapevine plants as substrate velocity at the dominant frequency (mm/s) and accordingly, three probability levels of successful mating communication were assigned to each point: »high«, velocity of mating signals > 0.01 mm/s, green circles; »median«, velocity of mating signals between 0.001 and 0.01 mm/s, blue circles; »low«, velocity of disruptive signals under 0.001 mm/s, pink circles. The latter is below the threshold level of signal detection of *S. titanus* (17). A mating pair of *S. titanus* is shown next to the grapevine plant (photo A. Lucchi).

In order to simulate a natural situation in a vineyard the potted grapevine plants were tied in a row to the grapevine supporting wire at various distances. On these plants the highest intensities of male calling signal were measured on the leaf which was vibrated with the pre-recorded calls (m = 1.45×10^−5^±0.56×10^−5^ mm/s), nevertheless, at almost all measuring points the recorded intensities were high enough to enable communication between the male and female (Figure 1) [17]. The mean substrate velocity measured from all other leaves was 2.19×10^−6^ ±1.37 mm/s.

**Figure 2:**
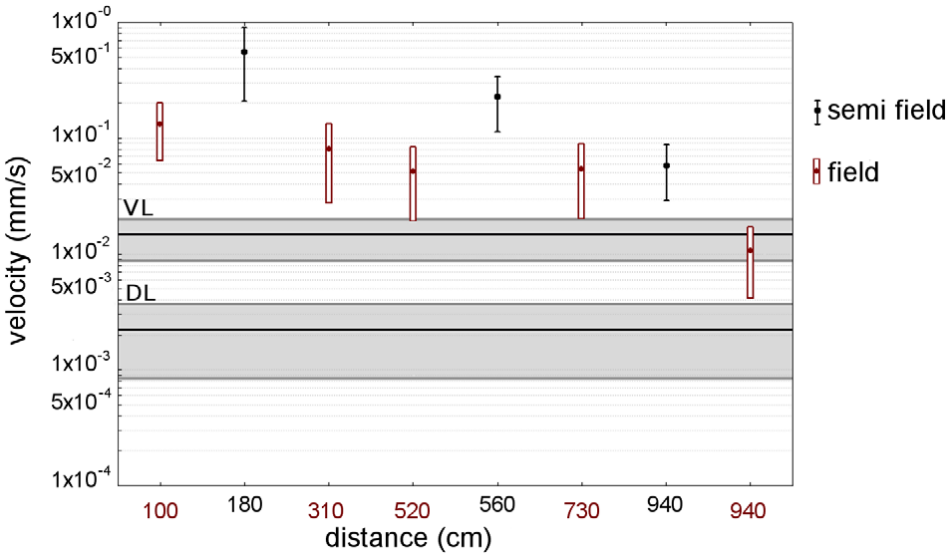
Maximum (mean±SD) substrate velocity (mm/s, logarithmic scale) of disruptive signal (DN) recorded in the frequency range 50-300 Hz from potted plants in semi-field conditions and from rooted grapevine plants in field. Semi-field and field recordings were made at three and five distances from a DN source, respectively (semi-field: black dots, distances 180 cm, 560 cm, 940 m; field: red dots, distances 100 cm, 310 cm, 520 cm, 730 cm, 940 cm). MCS played back into potted plants from a leaf showed highest substrate velocities within the same vibrated leaf (VL) range; a substantial decrease was found on all other leaves of the plant (distant leaves, DL). The transverse black lines represent the mean (±SD, gray areas) of maximum velocity of MCS of the VL or DL range.

The disruptive vibrational signals were applied to several potted plants simultaneously via the supporting wire up to 940 cm from the source of masking signals. An electromagnetic shaker was used to vibrate the wire with a pre-recorded *S. titanus* species-specific disruptive signal (disturbance noise) [13] and we determined the masking effect on male calling signal at several points along each plant. Although the ratio between the measured level of disruptive signal and male calling signal decreased with increasing distance of the plant from the shaker, even at 940 cm from the source, disruptive vibrational signals still masked male calling signals at every measured point (Figures 1 and 2).

**Figure 3:**
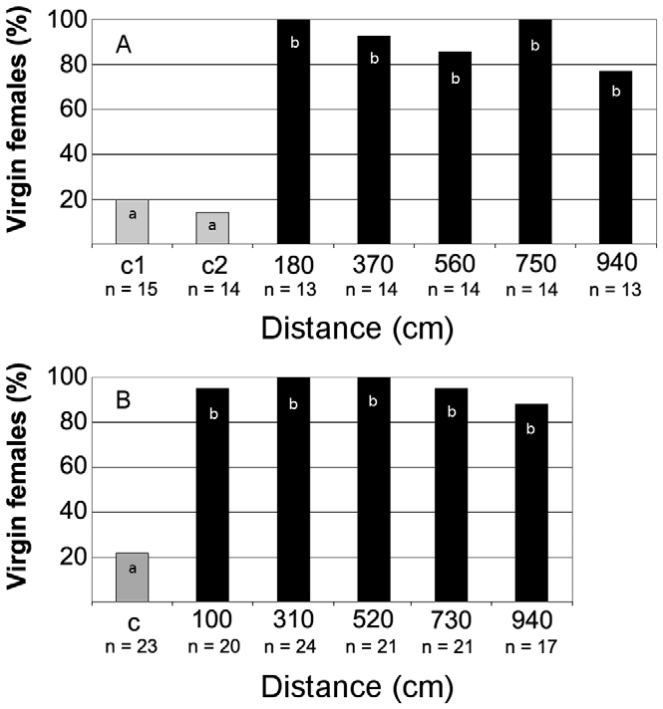
Number of virgin and mated females found on vibrated and non-vibrated grapevine plants. (A) Semi-field conditions with potted plants, (B) field trial in a vineyard. Black and gray bars show virgin females from plants at increasing distances from the source of disruptive signals and from control plants in the absence of these signals, respectively. Different letters indicate significant differences (P<0.0001) between treatments after G-test for contingency table (William’s corrected) followed by a Ryan multiple comparison of proportions test. The number of replicates (n) at each distance from the source of disruptive signals and for controls (c) is given.

Next, we assessed copulation success of *S. titanus* under simulated semi-field conditions as described above by comparing the number of eggs produced by females left with males overnight on vibrated and non-vibrated grapevine plants. In pairs that were placed on potted grapevine plants vibrated with disruptive signals, significantly more females remained virgin when pairs were put on vibrated plants (Figure 3A; G = 58.4, df = 6, P<0.0001) and no significant difference in copulation success at different distances was found.

### Mature vineyard

In a mature grape-producing vineyard, insect pairs were released overnight on plants positioned at similar distances as in semi-field trial. The last grapevine plant was positioned 940 cm away from the source of disruptive signals and the measured levels of disruptive signal were in the same intensity range as the naturally emitted *S. titanus* male calling signal (Figure 2). There was a significant difference in the number of virgin females between control and vibrated plants (Figure 3B; G = 119.7, df = 5, P<0.0001) but not between vibrated plants positioned at different distances.

## Discussion

Taken together, these results provide direct evidence that mating disruption based on playback of disruptive vibrational signals is an effective, environmentally friendly approach to manage insect pests. Although few females on vibrated plants placed at further distance from the source were mated, these matings could be explained by chance, as a result of call-fly behaviour [18] when males accidentally landed on the same leaf close to the females and when other potential factors like short-range chemical or visual cues may enable partner recognition. Taking into account the intensity loss of mating vibrational signal measured on distant leaves, in the presence of disruptive signals mating communication between pairs placed on different leaves seems unlikely. However, such accidental location of the female would be even less likely in the open field, where the movements of the male would not be limited to only few leaves or to the same shoot.

As in mating disruption based on pheromones, management of insect pests by disruptive vibrational signals does not eliminate pests from the system but can keep populations below an acceptable economic damage threshold [4]. Since delays in mating result in reduced female fecundity and fertility [19], long-term use of mating disruption can also decrease population levels of target pest species [3], [5]. Future work should reveal whether disruptive vibrational signals also affect other behaviours of target pests, such as feeding and oviposition, as well as whether they have negative effects on beneficial fauna. Parasitoids [20] and predators [21] use vibrational signals to locate their prey and masking signals could affect their localization ability. However, in our field trials spiders preying on *S. titanus* were a persistent problem and, potentially, visually-oriented predators like some spiders may be less affected.

Besides *S. titanus*, there are several other leafhopper and planthopper grapevine pests [22]-[25], including the vector of a lethal Pierce’s disease [6], against which this new tool for insect pest control could be implemented. Although in the current study we used *S. titanus* species-specific disruptive signals, it may be possible to synthesize a disruptive signal suitable for managing several pests simultaneously. As vibrational signalling is widespread among insects [14], mating disruption strategies for control of insect pests communicating via substrate-borne vibrational signals is likely to have wider application. Whiteflies are serious pests in greenhouses and vibrational signals are produced as part of their mating behaviour [26]. Vibrating large number of plants in the greenhouse may be easier than large scale field application for which additional work is needed to parameterise the effects of distance, and hence the spacing of vibrational sources, as well as potential interference of multiple sources of disruptive signals. Furthermore, it has been suggested that a monitoring trap, which combines pheromones and vibrational signals may provide a solution for pests like stink bugs that rely on bimodal communication [4]. In short term, the main challenge for application of vibrational mating disruption may be to convince growers, as well as policy makers, that this is a viable alternative to conventional plant protection tactics and that vibrating wires could reduce or replace the use of chemical pesticides. Moreover, in combination with novel monitoring techniques and as a part of an integrated high-tech crop protection system [27], mating disruption based on substrate-borne vibrational signals can provide an efficient pest management with low environmental impact that in the near future could transform many farming systems.

## Materials and Methods

### Insects

We collected *S. titanus* eggs from organic farms in Villazzano (Trento, Italy) and from them reared the adults used in the semi-field and field trials as described previously [13], [15], [17]. All experiments were made with sexually mature males and females that were at least 8 or exactly 10 days old, respectively [13].

### Signal transmission through grapevine plants

A *S. titanus* male calling song (MCS) used in transmission study was recorded with a laser vibrometer (PDV-100, Polytech GmbH, Waldbronn, Germany) in the laboratory with male singing at 0.5 cm distance from the recording point. To verify the characteristics of this signal, we compared it to the signals recorded and described previously [13], [17]. The disruption signal was a pre-recorded natural disruptive signal (also termed disturbance noise, DN) [13] recorded from a rival *S. titanus* male with the above mentioned laser vibrometer during rivalry encounters on a single grapevine leaf [13], [15]. An exemplar with the best signal-to-noise ratio was chosen from a library of recordings at Fondazione Edmund Mach (Italy).

Measurements in a semi-field setting were made outdoors at Pisa University (Italy) in July 2010. Five potted grapevine plants were pruned to have similar morphological characters (height 70-75 cm, two main branches, eight fully developed leaves). A supporting metal wire that commonly is used in vineyards was tied to the main stem of the plants, while the wire ends were fixed to concrete poles. The plants were placed in a row at distances 180 cm (plant 1), 370 cm (plant 2), 560 cm (plant 3), 750 cm (plant 4) and 940 cm (plant 5) from a newly designed electromagnetic shaker (power = 1 W, CBC Europe Ltd., Milano, Italy), through which the disruptive vibrational signal was applied (DN source) (Figure S3A). The shaker was driven by a lap-top computer via audio software Adobe Audition (version 3.0; Adobe Systems Inc.) and the amplitude of naturally emitted DN was amplified 20 times. MCS was applied to the lamina of the upper leaf via a conical rod attached to a mini-shaker (Type 4810; Brüel and Kjær Sound & Vibration A/S, Nærum, Denmark), driven from a computer via Adobe Audition and the amplitude was adjusted to the level of naturally emitted calls [17].

To study the signal transmission through the whole plant, small squares of reflective tape (n = 21) on which the laser beam was focused were placed on leaves (blades, veins and petioles) and along the stem (Figure S2). Vibrational signals were recorded with the above mentioned laser vibrometer and digitized with 48 kHz sample rate and 16-bit resolution, then stored directly onto a hard drive through Plug.n.DAQ (Roga Instruments, Waldalgesheim, Germany). The intensity of recorded signals was measured directly as maximum substrate velocity (mm/s) by Pulse 14 (Brüel and Kjær Sound & Vibration A/S). Only the spectral component within the natural range of *S. titanus* vibrational signals (50-300 Hz) (Figure S1) was analyzed, using a FFT window length of 400 points. MCS and DN were played back three times respectively for each measuring point on every plant and the velocity was then taken for the three pulses with highest amplitude, thus obtaining an average velocity from 9 pulses per measuring point. An average across the three plants was calculated for all points both from the vibrated leaf (VL: n = 7) and from all other leaves (distant leaves, DL: n = 8) (Figure S2). Points from the stem were excluded since *S. titanus* adults normally dwell on leaves. Our preliminary observations showed that the masking effect of DN on MCS was effective when the former was as high in intensity as the latter.

Field tests in a grape producing vineyard were conducted at Fondazione Edmund Mach (Italy) in July and August 2011. Mature rooted grapevine plants (height 1.5 m) grew in a row at distances 70 cm from each other with stems tied to a supporting metal wire. A MP3 driven electromagnetic shaker (EMS) used as source of disruptive signals (power = 1 W, CBC Europe Ltd., Milano, Italy) was attached to the wire and plants were chosen 100, 310, 520, 730 and 940 cm distant (Figure S3B). Disruptive signals were recorded as described above from four leaves/plant (two points/leaf), randomly chosen among those enclosed in the net sleeves, used for the mating disruption test (see below).

### Mating disruption

Experiments with live *S. titanus* in the semi-field setting of five potted grapevine plants, as described above, were conducted outdoors at Pisa University in July and August 2010. In addition, two potted grapevine plants of similar size tied to a non-vibrated wire were used as controls. Each plant was isolated in a transparent polyester cage (75×75×115 cm) (Bugdorm 2400 Insect Rearing Tent, MegaView Science Co., Ltd., Taichung, Taiwan) with closable openings to release and collect the insects. As a control, one grapevine plant from a neighbouring row without disruptive vibrations was used.

In field experiments, a shoot from the middle part of each plant (with approximately 20 leaves) was isolated in a nylon-netting sleeve (30×70 cm) (Bugdorm Insect Rearing Sleeves) with closable openings to release and collect the insects (Figure S4).

Since most mating activity in *S. titanus* occurs during twilight or during the night [13], all trials were made between 5 pm and 10 am the following day when insects were recollected from the cages/sleeves. In each overnight trial one virgin male and female *S. titanus* were put on separated leaves of each grapevine plant. When a male or a female could not be found or when one individual was dead, the replicate was discarded. Collected females were placed individually in rearing containers without access to egg laying sites and dissected 10 days later. Difference between the number of mated and virgin females in the treated plants and control plants was assessed with a G test in contingency table, after Williams’ correction. The G-test was followed by pair-wise comparisons between groups with Ryan’s test for multiple comparisons of proportions [28].

### Definition of virgin and mated S. titanus females

In preliminary experiments 10 days old females (n = 35) were placed together with males and observed until they copulated. Afterwards the females were kept for 10 days individually in rearing containers without suitable egg laying substrate. As a control, 20 days old virgin females (n = 35) were used. Shortly before a dissection, a living female was put in the freezer for 40 seconds before she was put in ethanol (70 %) under the stereomicroscope. Virgin females had on average 1.3 (±1.6) eggs, while mated females of the same age dissected 10 days after copulation had significantly higher number of eggs (13.4±3.7; n = 35; one-tailed unpaired t-test: t = −17.8, P<0.001). The minimum number of eggs found in the mated females was 7, while the maximum number of eggs in the virgin females was 6. Accordingly, we defined all females with 0-6 eggs as virgin and the females with >10 eggs as mated. As a safety limit, two females with 7-9 eggs were discarded. The eggs found in the virgin females were probably unfertilized and without the potential for development, as was suggested in the closely related species *Homalodisca vitripennis* (Hemiptera: Cicadellidae) [29].

## Supporting Information

Figure S1 **Oscillogram of a** ***Scaphoideus titanus*** **male calling song (MCS) (A) and of disturbance noise (DN) (B), both recorded on the same leaf, approximately 0.5 cm away from the male**. Power spectra of a male pulse (indicated with the asterisk in A) of MCS and of the whole DN sequence are shown in (C) and (D), respectively. MCS and DN are shown at natural magnification; for mating disruption trials the amplitude of DN was amplified 20 times.
(TIF)

Figure S2 **Schematic drawing of the measuring points on the grapevine plants used in the transmission experiment.** Abbreviations: RP, reference point; B, blade; V, vein; P, petiole; S, stem; MS, main stem. Yellow and pink dots indicate the points used to analyze the signal intensity of the Vibrated Leaf and the Distant Leaves, respectively. RP is in red. Points of the stem (in blue) were not included in the analysis.
(TIF)

Figure S3 **Experimental set-up of mating disruption trials in semi-field with potted plants enclosed in cages (A) and in a mature vineyard with shoots of the rooted plants enclosed in nylon netting sleeves (B).** The disruptive signals (DN) were emitted from an electromagnetic shaker (EMS) attached to the supporting wire. Recordings of vibrational signals were made with a laser vibrometer at 180 cm, 560 cm and 940 cm from EMS in semi-field and at all plants with sleeves in the vineyard. One insect pair was put in each cage/sleeve on grapevine plants at increasing distance from the source.
(TIF)

Figure S4 **Mature vineyard with shoots of grapevine plants isolated by sleeves (photo: V. Mazzoni).**
(TIF)

## References

[1] Greenfield MD (2002). Signalers and Receivers: Mechanisms and Evolution of Arthropod Communication..

[2] Gaston LK, Shorey HH, Saario SA (1967). Insect population control by the use of sex pheromones to inhibit orientation between the sexes.. Nature.

[3] Witzgall P, Kirsch P, Cork A (2010). Sex pheromones and their impact on pest management.. J Chem Ecol.

[4] Čokl A, Millar J, Ishaaya I, Horowitz AR (2009). Manipulation of insect signalling for monitoring and control of pest insects.. Biorational Control of Arthropod Pests.

[5] Ioriatti C, Lucchi A, Bagnoli B, Koul O, Cuperus GW, Elliot N (2008). Grape area wide pest management in Italy.. Areawide Pest Managements: Theory and Implementation.

[6] Redak RA, Purcell AH, Lopes JRS, Blua MJ, Mizell RF (2004). The biology of xylem fluid-feeding insect vectors of *Xylella fastidiosa* and their relation to disease epidemology.. Annu Rev Entomol.

[7] Weintraub PG, Beanland L (2006). Insect vectors of phytoplasmas.. Annu Rev Entomol.

[8] Janse JD, Obradovic A (2010). *Xylella fastidiosa* – its biology, diagnosis, control and risks.. J Plant Pathol.

[9] Dietrich H (2004). Phylogeny of the leafhopper subfamily Evacanthinae with a review of Neotropical species and notes on related groups (Hemiptera: Membracoidea: Cicadellidae).. Syst Entomol.

[10] Urban JM, Cryan JR (2007). Evolution of the planthoppers (Insecta: Hemiptera: Fulgoroidea).. Mol Phylogenet Evol.

[11] Čokl A, Virant-Doberlet M (2003). Communication with substrate-borne signals in small plant-dwelling insects.. Annu Rev Entomol.

[12] Mirandax (2006). Substrate-borne signal repertoire and courtship jamming by adults of *Ennya chrysura* (Hemiptera: Membracidae).. Ann Entomol Soc Am.

[13] Mazzoni V, Prešern J, Lucchi A, Virant-Doberlet M (2009). Reproductive strategy of the Nearctic leafhopper *Scaphoideus titanus* Ball (Hemiptera: Cicadellidae).. Bull Entomol Res.

[14] Cocroft RB, Rodriguez RL (2005). The behavioral ecology of insect vibrational communication.. BioScience.

[15] Mazzoni V, Lucchi A, Čokl A, Prešern J, Virant-Doberlet M (2009). Disruption of the reproductive behaviour of *Scaphoideus titanus* by playback of vibrational signals.. Entomol Exp Appl.

[16] Bressan A, Larrue J, Boudon Padieu E (2006). Patterns of phytoplasma-infected and infective *Scaphoideus titanus* leafhoppers in vineyards with high incidence of Flavescence dorée..

[17] Eriksson A, Anfora G, Lucchi A, Virant-Doberlet M, Mazzoni V (2011). Inter-plant vibrational communication in a leafhopper insect.. PLoS One.

[18] Hunt RE, Nault LR (1991). Roles of interplant movement, acoustic communication, and phototaxis in mate-location behaviour of the leafhopper *Graminella nigrifrons*.. Behav Ecol Sociobiol.

[19] Torres-Vila LM, Rodriguez-Molina MC, Stockel J (2002). Delayed mating reduces reproductive output of female European grapevine moth, *Lobesia botrana* (Lepidoptera: Tortricidae).. Bull Entomol Res.

[20] Laumann RA, Blassioli Moraes MC, Čokl A, Borges M (2007). Eavesdropping on sexual vibratory signals of stink bugs (Hemiptera: Pentatomidae) by the egg parasitoid *Telenomus podisi*.. Anim Behav.

[21] Virant-Doberlet M, King RA, Polajnar J, Symondson WOC (2011). Molecular diagnostics reveal spiders that exploit prey vibrational signals used in sexual communication.. Mol Ecol.

[22] Lenz MS, Isaacs R, Flore JA, Howell GS (2009). Vegetative growth responses of pinot gris (*Vitis vinifera* L.) grapevines to infestation by potato leafhoppers (*Empoasca fabae* Harris).. Am J Enol Vitic.

[23] Costello MJ (2008). Regulated deficit irrigation and density of *Erythroneura* spp. (Hemiptera: Cicadellidae) on grape.. J Econ Entomol.

[24] Mazzoni V, Lucchi A, Ioriatti C, Virant-Doberlet M, Anfora G (2010). Mating behaviour of *Hyalesthes obsoletus* (Hemiptera: Cixiidae).. Ann Entomol Soc Am.

[25] Pavan F, Picotti P (2009). Influence of grapevine cultivars on the leafhopper *Empoasca vitis* and its egg parasitoids.. BioControl.

[26] Kanmiya K, Drosopoulos S, Claridge MF (2006). Mating behaviour and vibratory signals in Whiteflies (Hemiptera: Aleyrodidae).. Insect Sounds and Communication: Physiology, Behaviour, Ecology and Evolution.

[27] Clay J (2011). Freeze the footprint of food.. Nature.

[28] Ryan TA (1960). Significance test for multiple comparison of proportions, variances and other statistics.. Psychol Bull.

[29] Al-Wahaibi AK, Morse JG (2009). Egg morphology and stages of embryonic development of the glassy-winged sharpshooter (Hemiptera: Cicadellidae) Ann Entomol Soc Am.

