# Correction: Exploitation of Insect Vibrational Signals Reveals a New Method of Pest Management

**DOI:** 10.1371/journal.pone.0100029

**Published:** 2014-06-06

**Authors:** The PLOS ONE Staff

There are errors in the funding statement. The correct funding statement is as follows: This research was supported by the Autonomous Province of Trento (Accordo di Programma 2010), Funding Research Programme P1-0255 and Research Project V4-0525 by Slovenian Research Agency, Fondi Ateneo of Pisa University, the European Union Seventh Framework Programme (FP7/ 2007-2013) under the grant agreement n°265865, and CBCEurope Ltd. (Milano, Italy). The funders had no role in study design, data collection and analysis, decision to publish, or preparation of the manuscript.

## References

[1] Eriksson A, Anfora G, Lucchi A, Lanzo F, Virant-Doberlet M, et al (2012) Exploitation of Insect Vibrational Signals Reveals a New Method of Pest Management. PLoS ONE 7(3): e32954 doi:10.1371/journal.pone.0032954 22457726 10.1371/journal.pone.0032954 PMC3310055

