# Supplementary figures and images for: Exploitation of Insect Vibrational Signals Reveals a New Method of Pest Management
Source: PLoS One. 2012 Mar 21;7(3):e32954. doi: 10.1371/journal.pone.0032954 (PMC3310055; doi:10.1371/journal.pone.0032954)

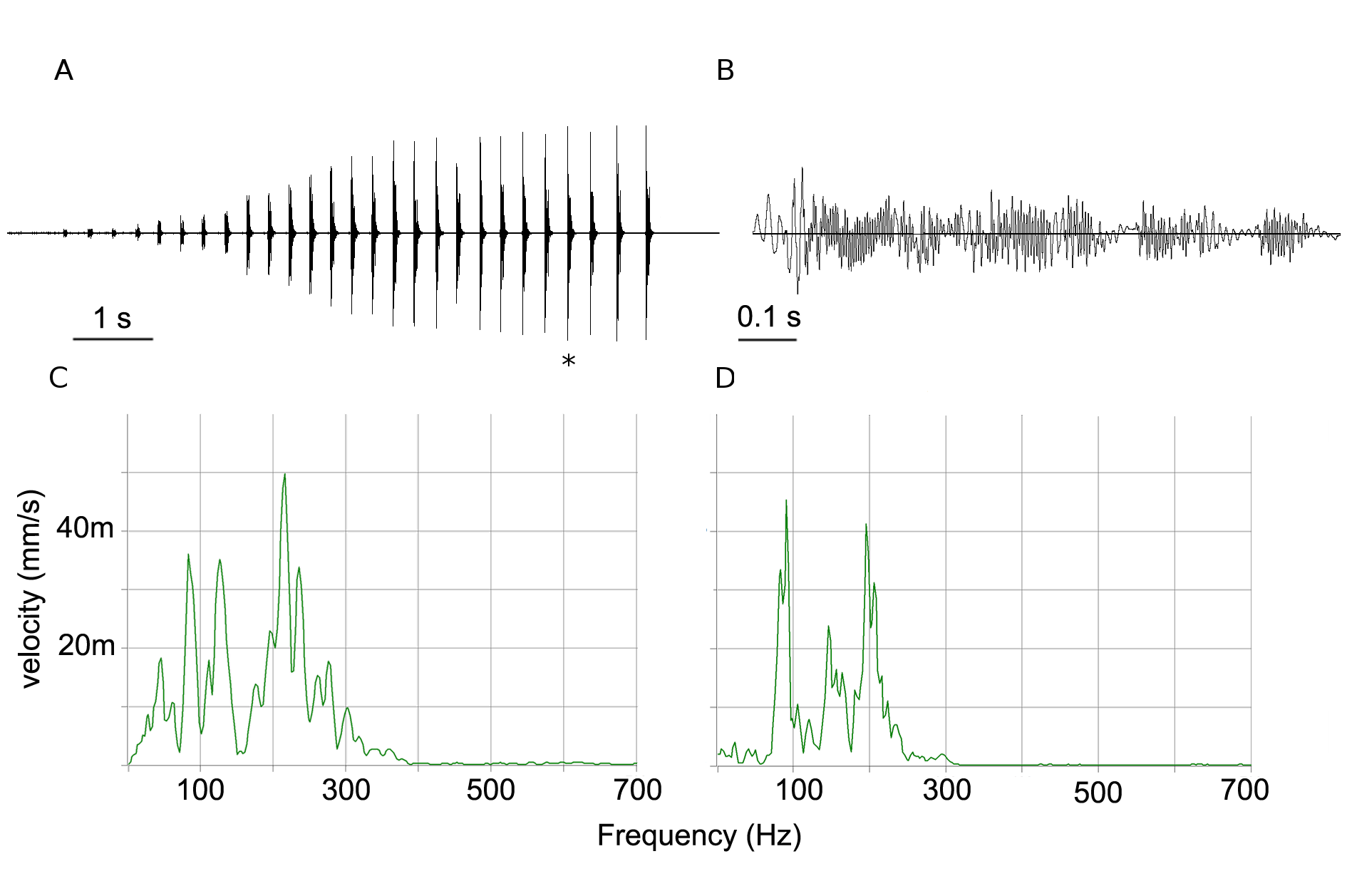

Supplement: Figure S1 — Oscillogram of a Scaphoideus titanus male calling song (MCS) (A) and of disturbance noise (DN) (B), both recorded on the same leaf, approximately 0.5 cm away from the male. Power spectra of a male pulse (indicated with the asterisk in A) of MCS and of the whole DN sequence are shown in (C) and (D), respectively. MCS and DN are shown at natural magnification; for mating disruption trials the amplitude of DN was amplified 20 times. (TIF) [file pone.0032954.s001.tif]

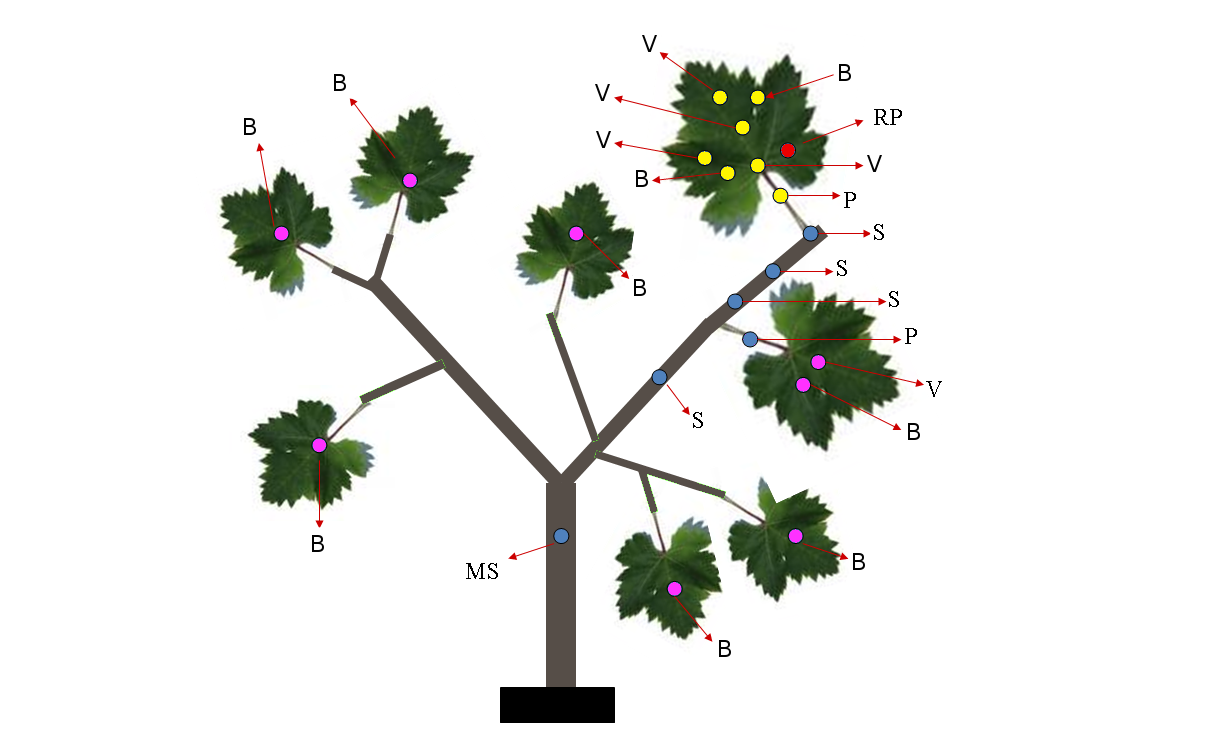

Supplement: Figure S2 — Schematic drawing of the measuring points on the grapevine plants used in the transmission experiment. Abbreviations: RP, reference point; B, blade; V, vein; P, petiole; S, stem; MS, main stem. Yellow and pink dots indicate the points used to analyze the signal intensity of the Vibrated Leaf and the Distant Leaves, respectively. RP is in red. Points of the stem (in blue) were not included in the analysis. (TIF) [file pone.0032954.s002.tif]

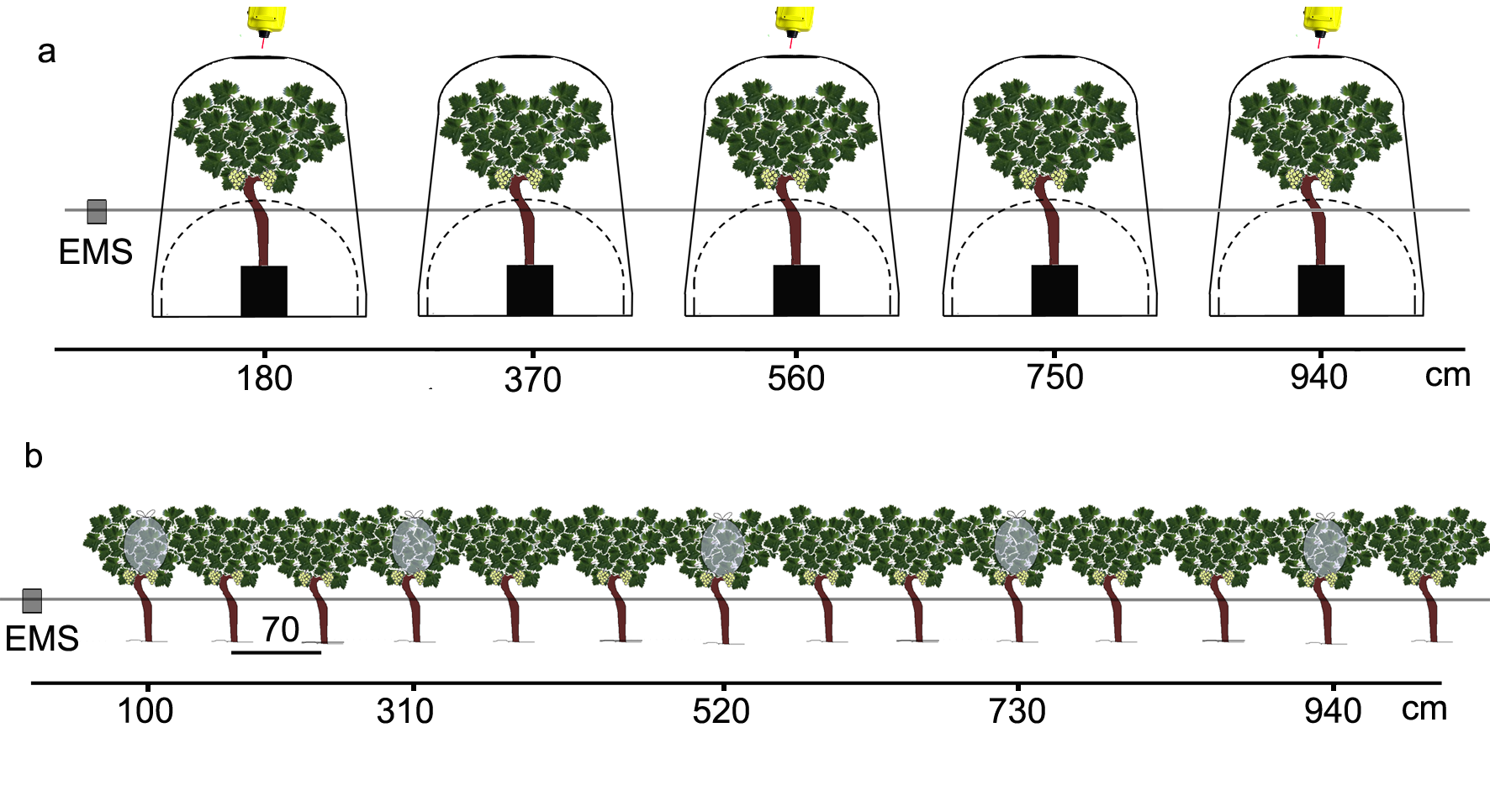

Supplement: Figure S3 — Experimental set-up of mating disruption trials in semi-field with potted plants enclosed in cages (A) and in a mature vineyard with shoots of the rooted plants enclosed in nylon netting sleeves (B). The disruptive signals (DN) were emitted from an electromagnetic shaker (EMS) attached to the supporting wire. Recordings of vibrational signals were made with a laser vibrometer at 180 cm, 560 cm and 940 cm from EMS in semi-field and at all plants with sleeves in the vineyard. One insect pair was put in each cage/sleeve on grapevine plants at increasing distance from the source. (TIF) [file pone.0032954.s003.tif]

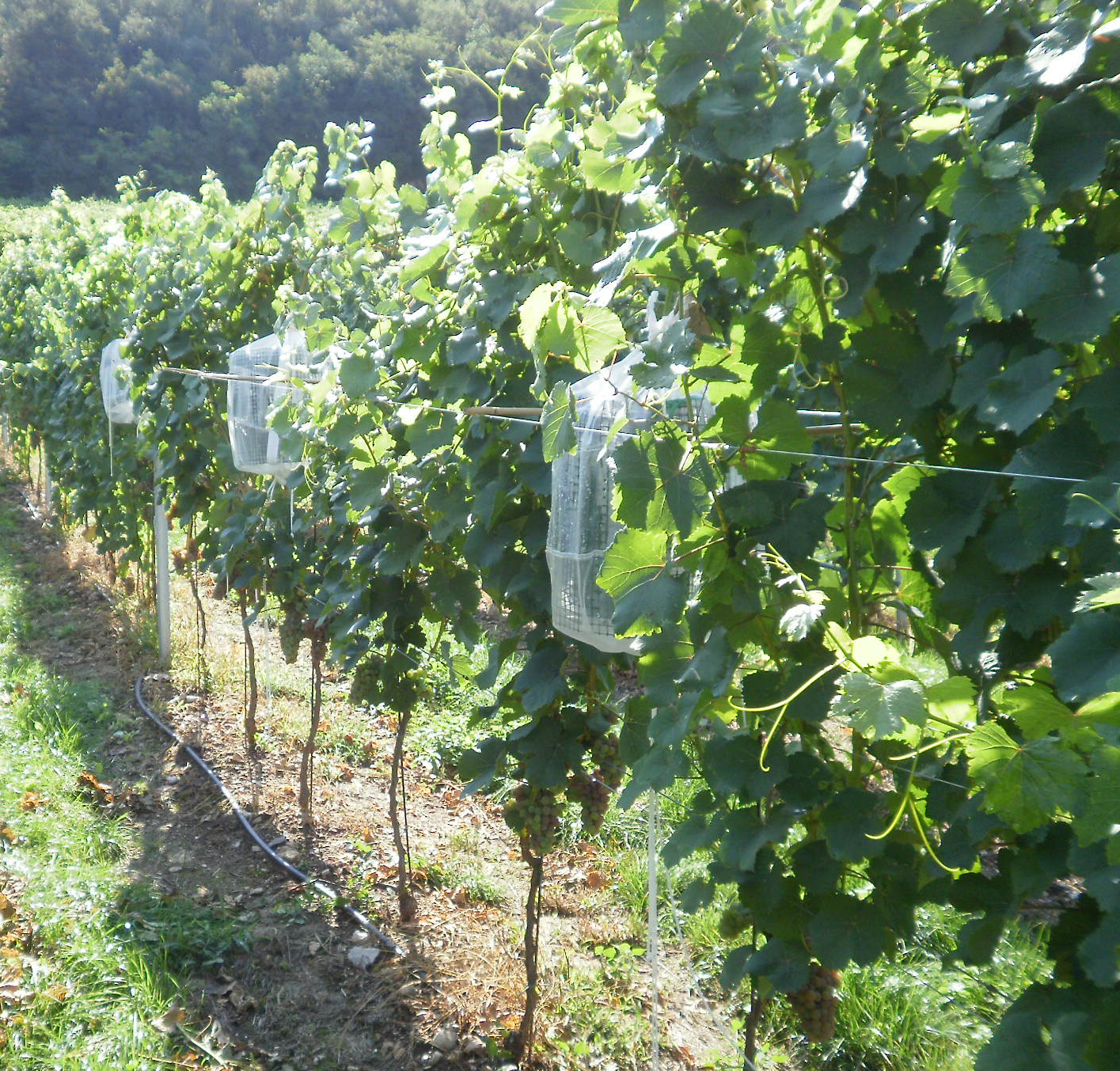

Supplement: Figure S4 — Mature vineyard with shoots of grapevine plants isolated by sleeves (photo: V. Mazzoni). (TIF) [file pone.0032954.s004.tif]
